# Development of a Selection Method for Discovering Irreversible (Covalent) Binders from a DNA-Encoded Library

**DOI:** 10.1177/2472555218808454

**Published:** 2018-11-01

**Authors:** Zhengrong Zhu, LaShadric C. Grady, Yun Ding, Kenneth E. Lind, Christopher P. Davie, Christopher B. Phelps, Ghotas Evindar

**Affiliations:** 1 GlaxoSmithKline, Cambridge, Massachusetts, USA

**Keywords:** DNA-encoded library technology, DEL, DNA-encoded chemical libraries, covalent inhibitors, irreversible inhibitors, affinity selections, selection of covalent binders

## Abstract

DNA-encoded libraries (DELs) have been broadly applied to identify chemical probes for target validation and lead discovery. To date, the main application of the DEL platform has been the identification of reversible ligands using multiple rounds of affinity selection. Irreversible (covalent) inhibition offers a unique mechanism of action for drug discovery research. In this study, we report a developing method of identifying irreversible (covalent) ligands from DELs. The new method was validated by using 3C protease (3CP) and on-DNA irreversible tool compounds (rupintrivir derivatives) spiked into a library at the same concentration as individual members of that library. After affinity selections against 3CP, the irreversible tool compounds were specifically enriched compared with the library members. In addition, we compared two immobilization methods and concluded that microscale columns packed with the appropriate affinity resin gave higher tool compound recovery than magnetic beads.

## Introduction

In recent years, the primary focus in drug discovery has been on reversible inhibitors, with limited attention paid to irreversible (covalent) inhibitors. A core reason for this may be due to the lack of appropriate screening collection compounds in some pharmaceutical companies for irreversible inhibitors. We believe DNA-encoded libraries (DELs) can provide an answer to this challenge and open up an additional avenue to take advantage of the therapeutic benefits of covalent inhibitors. The high biochemical efficiency of irreversible inhibitors may translate into lower dose and reduced off-target effects. Uncoupling pharmacokinetics and pharmacodynamics and prolonging the duration of action by irreversible inhibition may result in less frequent drug dosing. Many approved drugs exploit this opportunity.^1–4^

**Figure 1.**
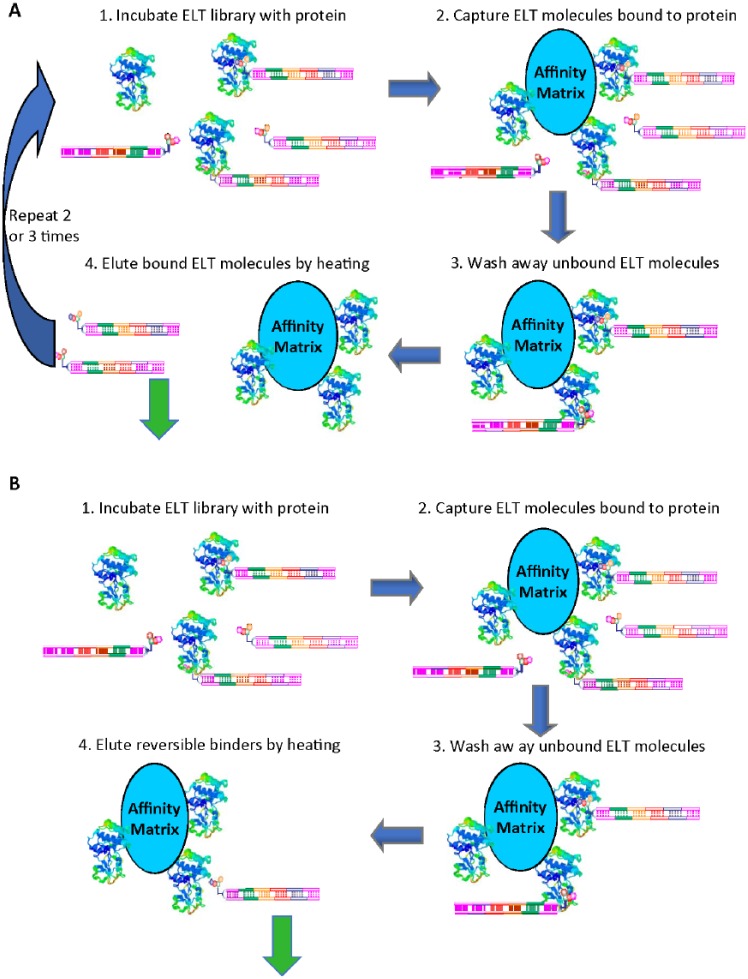
(**A**) Typical DEL affinity selection for identifying reversible binders to target proteins. (**B**) DEL affinity selection method for identifying irreversible binders.

DEL technology is a platform for identifying small-molecule ligands to protein targets using affinity selection of DNA-tagged combinatorial libraries.^5–15^ Reported efforts to use encoded libraries to identify irreversible binders have been restricted to single-step syntheses; these include a DNA-encoded microarray of 625 chemical fragments,^16^ a peptide nucleic acid (PNA)-encoded microarray of combinations of 100 amino acids and 100 Michael acceptors,^17^ and two self-assembling libraries of 265 and 559 members.^18^ None of these applications exploit the diversity advantage of typical DNA-encoded compound libraries made by multistep combinatorial synthesis. Affinity selection methods commonly used for DELs are described in **Figure 1A**. After each round of selection, reversible binders are eluted from the target protein by thermal denaturation, and then used in the next round of selection; however, irreversible binders would not be expected to elute unless they are labile under the elution conditions. Although this selection process is very effective at finding reversible binders, it is not suited for the identification of irreversible binders. To identify irreversible binders from a DEL, we redesigned the DEL affinity selections with only one round of selection (**Fig. 1B**). After removing reversible binders by heat elution, DEL molecules irreversibly bound to target protein immobilized on affinity matrix are directly amplified by PCR on the beads for sequencing.

**Figure 2.**
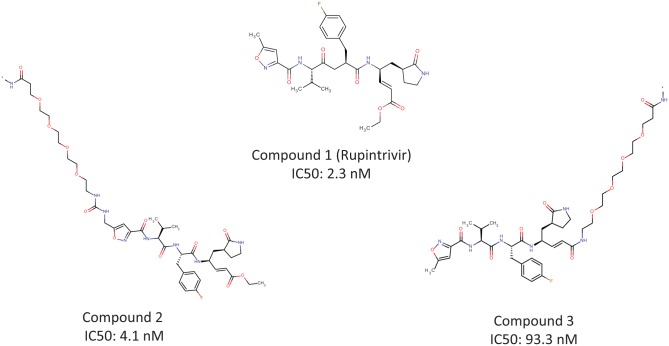
Tool compounds for 3CP selections.

3C protease (3CP) was selected as a target to explore this strategy as we had enough experience with the target protein and the tool compounds with well-understood structure–activity relationship (SAR). 3CP exists in many viruses (picornavirus, coronavirus, norovirus, etc.) and plays an essential role in the viral life cycle.^19–21^ Inhibition of 3CP may lead to potential treatments for viral-related diseases, for example, the common cold. Rupintrivir is a known, potent, irreversible (covalent) inhibitor of 3CP. DNA tags were conjugated with rupintrivir at two distinct positions (**Fig. 2**) to generate the on-DNA tool compounds used in this study of selection methods for irreversible inhibitors. The new method was validated by significantly enriching the irreversible tool compounds after spiking them into a DEL compound library at the same concentration as individual library members. This method of DEL affinity selection offers an enabling tool for challenging therapeutic targets.

## Materials and Methods

### Synthesis of On-DNA Tool Compounds of Human Rhinovirus (HRV) 3CP

We linked DNA to rupintrivir (compound **1**) at two distinct positions (**Fig. 2**). For compound **2**, the amine derivative of rupintrivir was linked to DNA via a urea linkage.^22,23^ The amino-functionalized DNA was reacted with PNP-Cl to form isocyanate, which was further reacted with an amine derivative of rupintrivir to yield the on-DNA tool. For compound **3**, the acid form of rupintrivir was acylated with DNA through HATU activation, which led to another on-DNA tool (SI). The concentration of each on-DNA tool compound with one unique DNA tag was determined by UV absorption at 260 nm. Both on-DNA tools were purified by reverse-phase high-performance liquid chromatography (HPLC) and then tested in 3CP enzyme activity assay (**Fig. 2**). Compound **2** was more potent than compound **3**, and the potency of the former was close to that of rupintrivir without a DNA tag.

### DEL Selection for Irreversible Binders Using Affinity Resins

Following the PhyTip MEA Purification System manual (Phy Nexus, San Jose, CA; https://www.phynexus.com), protocols and methods were created with PhyNexus Controller software installed on the MEA System. Tool compounds alone (10^6^–10^9^ molecules) or a DEL (2.5 nmol, 7.6 million members, approximately 2 × 10^8^ molecules of each library member) with 2 × 10^8^ molecules each of the tool compounds spiked in were incubated with His-tagged 3CP (5 µg) in 60 µL of selection buffer (50 mM Tris-HCl [pH 7.5], 150 mM NaCl, 0.1% Tween-20, 0.1 mg/mL sheared salmon sperm DNA [Ambion]) for 1 h at room temperature. A mixture of library and tool compounds without 3CP were also incubated under the same conditions as a negative control to assess background binding of DNA-encoded molecules to the affinity resin. The MEA System was used to capture the protein–library mixture on affinity resin tips, which were then washed five times with 100 µL of selection buffer to remove unbound DEL molecules. To elute reversible molecules, resins were incubated in 60 µL of selection buffer at 80 °C for 10 min. After cooling down to room temperature for 5 min, a scalpel was used to cut open the caps of affinity resin tips and a fine dosing syringe was used to blow resins into 1.5 mL microcentrifuge tubes. Water (100 µL) was added and vortexed for 10 s, and then 1 µL was used to run quantitative PCR (qPCR) on a Roche LightCycler 480 (Penzberg, Germany). Based on the copy number of DNA-encoded molecules determined by qPCR, an appropriate number of PCR cycles was selected for the amplification and addition of DNA sequences compatible with Illumina sequencing flow cells. PCR output was purified using Agencourt AMPure XP SPRI beads (Agencourt, Danvers, MA) according to the manufacturer’s instructions, and then quantitated on an Agilent BioAnalyzer (Santa Clara, CA) using a high-sensitivity DNA kit. The final concentration of amplicon for each sample was between 3 and 40 nM. Each sample was loaded onto an Illumina GAII or HiSeq sequencer (San Diego, CA) to generate ~20 million sequences per sample. Each DNA tag included a random N12 region that acts as a unique molecular identifier for every library molecule to discriminate binding events from amplification events in the sequencing data. Sequences with identical N12 regions were counted as a single binding event. A detailed description of general DEL selection steps can be found in Goodnow.^15^ Based on the sequence information obtained, copy counts were determined for all library members and the tool compounds.

### DEL Selection for Irreversible Binders by Using Magnetic Beads

His-tagged 3CP (5 µg) and DEL/tool compound mixtures were prepared as above and incubated in 60 µL of selection buffer (50 mM Tris-HCl [pH 7.5], 150 mM NaCl, 0.1% Tween-20, 0.1 mg/mL sheared salmon sperm DNA [Ambion]) for 1 h at room temperature. A mixture of library and tool compounds without 3CP were also incubated under the same condition as a negative control to assess background binding of the DNA-encoded molecules to affinity resins. HisPur Ni-NTA magnetic beads (25 µL, Thermo Scientific 88832, Waltham, MA) were washed once with 500 µL of selection buffer. The protein–library mixture was added to the magnetic beads, vortexed for 10 s, and then placed into a DynaMag 2 magnet to collect the magnetic beads against the side of the tube and discard the supernatant. After the magnetic beads were washed once with 1 mL of selection buffer, 100 μL of selection buffer was added and vortexed for 10 s and then incubated at 95 °C for 10 min on a Thermomixer (Eppendorf, Westbury, NY) with 1000 rpm mixing. The magnetic beads were put on ice for 10 min before being placed into a DynaMag 2 magnet to remove the supernatant, which contained reversible binders. After 100 μL of water was added and vortexed for 10 s, 1 µL was withdrawn and used to run qPCR on a Roche LightCycler 480. Based on the copy number of DNA-encoded molecules determined by qPCR, an appropriate number of PCR cycles was selected for the amplification and addition of DNA sequences compatible with Illumina sequencing flow cells. PCR output was purified, sequenced, and analyzed using the same methods as for the PhyTip resin samples.

## Results and Discussion

**Figure 3.**
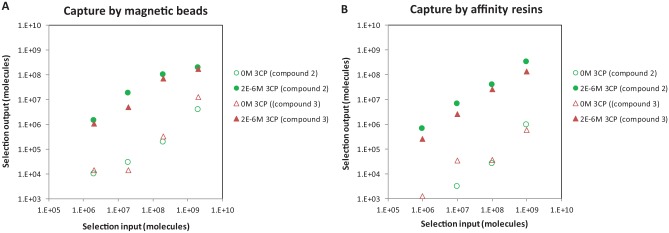
Evaluation of the selection method with on-DNA tool compounds. Selection input: amount of tool compound molecules added before affinity selection, individually determined by qPCR. Selection output: tool compound molecules (unique sequences) in the mixture determined by sequencing. The copy number of individual members from the library was counted based on sequencing data without manipulation. Since the sequencing primer sequence was only added during amplification after selection, qPCR using primers for constant regions in the DEL molecules had to be used to quantify selection inputs, while sequencing was used to quantify selection outputs.

The new selection method designed for irreversible (covalent) binders (**Fig. 1B**) was initially tested with the on-DNA tool compounds (compounds **2** and **3**) at different input concentrations to determine the minimum input for selections. Each compound was tagged with four different DNA tags to encode both the input concentration and compound ID. The encoded samples were then pooled for a single selection experiment. Two capture methods were used: magnetic beads and microscale affinity resin columns. After PCR amplification and sequencing, the number of copies detected for each DNA tag was counted and plotted in **Figure 3**. Selection output correlated very well with selection input of tool compounds with a signal window of about 2 orders of magnitude between 2 µM and 0 µM of 3CP. Copy counts for compound **2** were greater than those for compound **3**, correlating with the enzyme assay data.

**Figure 4.**
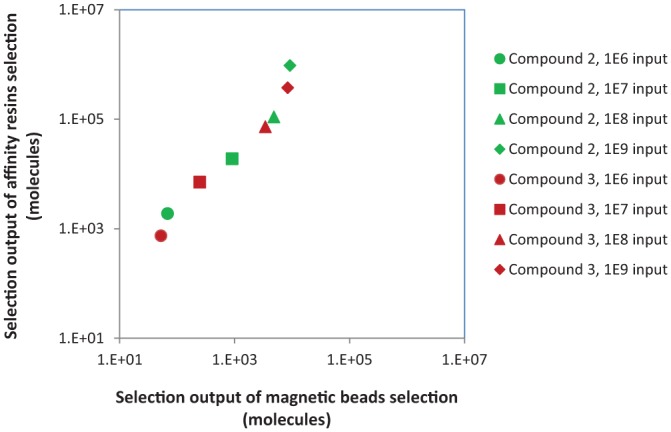
Comparison of capture methods between microscale affinity columns and magnetic beads for the new selection method. Two on-DNA tool compounds were used with four different input amounts. Each data point represents either different compounds or a different input amount. Copy counts of individual members from the library were obtained based on sequencing data without manipulation.

**Figure 4** shows a comparison of capture methods between affinity resins and magnetic beads. Under all conditions, the affinity resins gave a higher recovery of the input sample copies. Whether this result can be extended to other targets remains to be explored.

**Figure 5.**
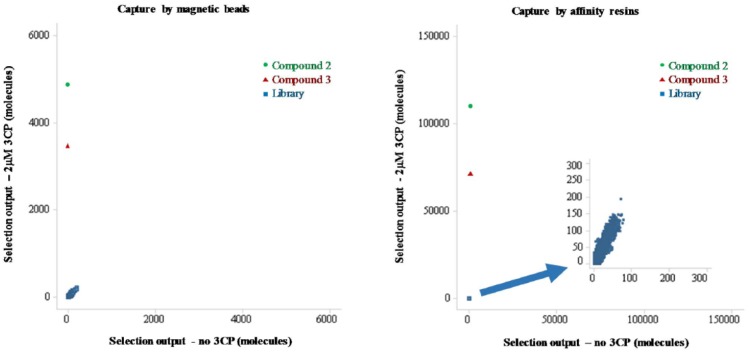
On-DNA tool compounds were spiked into a DEL of 7.6 million members at the same concentration as individual library members and then tested with the new selection method. After selection, 6.3 million compounds were detected by sequencing from the magnetic beads, and 6.2 million compounds were detected by sequencing from the PhyTip affinity resin. Some library molecules were detected in the presence and absence of 3CP, indicating background binding to the affinity matrices. Enrichment of the tool compounds was significantly above the diagonal line from background binding, indicating that these were specifically enriched by binding to 3CP.

In order to determine if this method could be used to identify irreversible binders from a diverse DEL, selections were run on the two on-DNA tool compounds spiked into a conventional DEL with a diversity of 7.2 million unique compounds at the same concentration as individual compounds in that library. The copy number of on-DNA tool compounds and library compounds are plotted in **Figure 5**. The tool compounds clearly showed specific enrichment well above the reversible library compounds with both capture methods when 2 µM 3CP was used, but low background signal when no 3CP was present. Comparing the two capture methods, copy counts for the tool compounds with the microscale affinity columns are higher than those with the magnetic bead, as is the enrichment compared with the control library members.

DEL affinity selections have proven to be capable of identifying active compounds for many therapeutic targets.^5–15^ However, when multiple rounds of selection are used, active compounds discovered from DELs are likely to be reversible (noncovalent) ligands. In this study, we designed and developed a DEL affinity selection method for identifying irreversible (covalent) binders. On-DNA tool compounds were synthesized and used for optimization and validation. The selection output of tool compounds correlated very well with the selection input and compound activity. Between two capture methods tested in this study, microscale affinity columns appeared to be a better option than magnetic beads in terms of total tool compound recovery and signal-to-noise ratio (**Figs. 4** **and** **5**). When tool compounds were spiked in a DEL at working library concentrations, they could be clearly identified after selection. This selection method offers an effective tool for discovering irreversible (covalent) active compounds for therapeutic targets.

## Supplemental Material

DS_DISC808454 – Supplemental material for Development of a Selection Method for Discovering Irreversible (Covalent) Binders from a DNA-Encoded Library Click here for additional data file. Supplemental material, DS_DISC808454 for Development of a Selection Method for Discovering Irreversible (Covalent) Binders from a DNA-Encoded Library by Zhengrong Zhu, LaShadric C. Grady, Yun Ding, Kenneth E. Lind, Christopher P. Davie, Christopher B. Phelps and Ghotas Evindar in SLAS Discovery
