# Supplementary material for: Development of a Selection Method for Discovering Irreversible (Covalent) Binders from a DNA-Encoded Library
Source: SLAS Discov. 2018 Nov 1;24(2):169–74. doi: 10.1177/2472555218808454 (PMC7221453; doi:10.1177/2472555218808454)

HATU Acylation

One hundred ten nanomoles of oligo was diluted to 1 mM in 250 mM sodium borate buffer (pH 9.4). Solutions of (*S*,*E*)-4-((*S*)-3-(4-fluorophenyl)-2-((*S*)-3-methyl-2-(5-methylisoxazole-3-carboxamido)butanamido)propanamido)-5-((*S*)-2-oxopyrrolidin-3-yl)pent-2-enoic acid (2.03 mg, 22 µl, 200 mM), DIPEA (31.86 mg, 1.232 mL, 200 mM), and HATU (19.23 mg, 253 µL, 200 mM) were prepared in chilled DMA (stored at 4 °C for 20 min). Forty equivalents of acid solution (22 µL) was added with DIPEA solution (22 µL), followed by 22 µL of the HATU solution. The solution was mixed by vortex and stored at 4 °C for 5 min. Then all 66 µL of the premixed solution was added to the oligo solution. The reaction was allowed to proceed at room temperature overnight.

The reaction mixture was added to 20 µL of 5 M NaCl and 440 µL of cold ethanol to crash out the DNA. After being stored at –80 °C for 2 h, the crashed reaction was spun down at 10,000 rpm and 4 °C for 1 h. After removing the supernatant, the resulting pellet was lyophilized until dry.

QPCR amplification curve and standard curve for calibration.


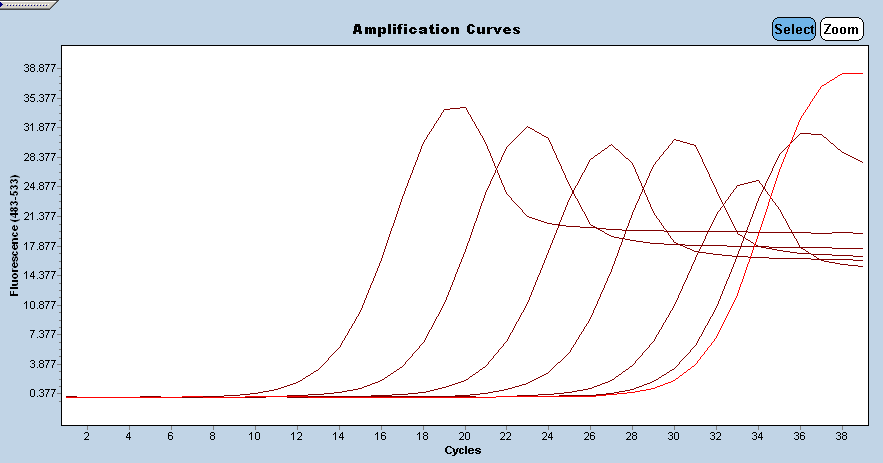


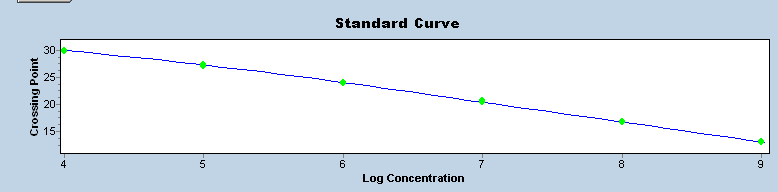

Supplement: DS_DISC808454 – Supplemental material for Development of a Selection Method for Discovering Irreversible (Covalent) Binders from a DNA-Encoded Library [file DS_DISC808454.docx]
